# Auxin regulated metabolic changes underlying sepal retention and development after pollination in spinach

**DOI:** 10.1186/s12870-021-02944-4

**Published:** 2021-04-06

**Authors:** Mahpara Fatima, Xiaokai Ma, Ping Zhou, Madiha Zaynab, Ray Ming

**Affiliations:** 1 College of Agriculture, FAFU and UIUC-SIB Joint Center for Genomics and Biotechnology, National Sugarcane Engineering Technology Research Center, Fujian Provincial Key Laboratory of Haixia Applied Plant Systems Biology, Fujian Agriculture and Forestry University, Fuzhou, 350002 Fujian China; 2 Department of Plant Biology, University of Illinois at Urbana-Champaign, Urbana, IL 61801 USA

**Keywords:** Pollination, Sepal development, Auxin biosynthesis, Spinach

## Abstract

**Background:**

Pollination accelerate sepal development that enhances plant fitness by protecting seeds in female spinach. This response requires pollination signals that result in the remodeling within the sepal cells for retention and development, but the regulatory mechanism for this response is still unclear. To investigate the early pollination-induced metabolic changes in sepal, we utilize the high-throughput RNA-seq approach.

**Results:**

Spinach variety ‘Cornel 9’ was used for differentially expressed gene analysis followed by experiments of auxin analog and auxin inhibitor treatments. We first compared the candidate transcripts expressed differentially at different time points (12H, 48H, and 96H) after pollination and detected significant difference in Trp-dependent auxin biosynthesis and auxin modulation and transduction process. Furthermore, several auxin regulatory pathways i.e. cell division, cell wall expansion, and biogenesis were activated from pollination to early developmental symptoms in sepals following pollination. To further confirm the role auxin genes play in the sepal development, auxin analog (2, 4-D; IAA) and auxin transport inhibitor (NPA) with different concentrations gradient were sprayed to the spinach unpollinated and pollinated flowers, respectively. NPA treatment resulted in auxin transport weakening that led to inhibition of sepal development at concentration 0.1 and 1 mM after pollination. 2, 4-D and IAA treatment to unpollinated flowers resulted in sepal development at lower concentration but wilting at higher concentration.

**Conclusion:**

We hypothesized that sepal retention and development might have associated with auxin homeostasis that regulates the sepal size by modulating associated pathways. These findings advanced the understanding of this unusual phenomenon of sepal growth instead of abscission after pollination in spinach.

**Supplementary Information:**

The online version contains supplementary material available at 10.1186/s12870-021-02944-4.

## Background

Pollination inaugurates global changes in gene expression pattern that leads to sepal and petal abscission [1]. However, in a few plant families, the protracted presence of sepal whose actual function is in pollen import and export suggested that following pollination, they are adaptive for new functions not related to pollination. For example, in *Helleborus foetidus* and *Paris polyphylla*, the green persistent sepals provide assimilates for the developing seeds and fruits [2, 3]. Sepal growth is also observed in various species of Asteraceae, Labiatae, Dilleniaceae, and Malvaceae after pollination, where it takes part in fruit development [4]. However, largely expanded sepals in *Physalis angulate* enhance plant fitness by providing herbivore protective function against parasitoids to the caterpillars that feed on the fruits enclosed within these sepals [5]. Keeping in view the post-pollination functions of sepal persistence, it is worthy to explore the regulatory mechanism behind its retention and growth after pollination.

Plant hormones homeostasis is involved in the onset of floral organ abscission. In ethylene-sensitive flowers, the first visible sign of senescence is accompanied by a transient and sudden rise of ethylene production [6]. Whereas ethylene triggers abscission, auxin appears to reduce the sensitivity of abscission cells to ethylene and thus prevent abscission [7, 8]. Exogenous application of auxin was found to delay or prevent flower abscission in apple [9], cherry, Phaseolus [10], cotton [11], and Geraldton wax flowers [12]. In *Cleome hassleriana*, the floral abscission was partially regulated by anthers, which had this effect by virtue of their continued auxin production [13]. Abscission of unfertilized flowers occurred due to low endogenous auxin biosynthesis in the ovary and is delayed or prevented by exogenous IAA treatment [14]. Recent advancements in the understanding of auxin biosynthesis, transport, and metabolism established that concentration gradient is a driving force for organogenesis and patterning, designating auxin as a plant morphogen [15]. However, a little is known about auxin effect on sepal development and retention control.

Spinach (*Spinacia oleracea*) is a vegetable plant, native to central and western Asia, and belongs to the family Amaranthaceae. Spinach has three reproductive systems: i) dioecy, ii) monoecy, and iii) hermaphroditism. Dioecious female spinach (XX) flowers have 4–5 stigmas and large sepals that persist and encase the seeds, whereas dioecious male spinach (XY) flowers have four stamens. The small sepals of male flowers are hardly visible after anthesis. Dioecy and monoecy are common, while hermaphroditism is rare. We inspected several Chinese spinach cultivars but none of them shows hermaphroditic characteristics. Hermaphrodite plants look like male plants with only one or a few hermaphrodite flowers, and it is difficult to differentiate between a style and a filament after the anther falls off. On the other hand, in monoecious (with male and female flowers on the same plant) plants, the female flower sepals grow the same as the dioecious female after pollination. This observation justified using dioecious female plants as an experimental system to study this phenomenon. Interestingly, persistent green sepals of female flower contribute to the plant’s fitness in terms of seed protection (Figure S1A, B). Pollination and fertilization trigger the expansion of the green sepals with the developing seeds, but it is unclear what metabolic changes resulting in the noticeable increase in sepal size.

This study was designed to profile early changes in gene regulation that modify spinach sepal size following pollination, to identify the regulatory metabolic changes underlying unusual sepal retention and development. Data from this study will provide a valuable addition to the molecular resources for spinach. This study will also guide the future selection of candidate genes for delaying flower senescence or promoting fruit sets by extending sepal longevity in other plant species.

## Results

### Pollen tube growth and 1st sampling stage

The characterization of pollen tube growth and its visual developmental symptoms in spinach female flowers were conducted to identify the best time point for 1st stage RNA-seq library construction. The aim was to find metabolic pathways involved in very early pollination-induced developmental changes within sepal after pollination. As sepals grow with the developing seeds after pollination, so time point (1st stage of sepal development) just before fertilization was desired. For this, pollen tube growth was measured at various times 0, 10, 12, 14 h after pollination (HAP). It was observed that pollen tube maintained a relatively steady, non-linear growth, reached at the end of ovule after 12 HAP but before 14 HAP (Figure S1C). So, 12 HAP was considered the 1st time point for library construction.

### Transcriptome assembly and differentially expressed genes analysis

**Fig. 1:**
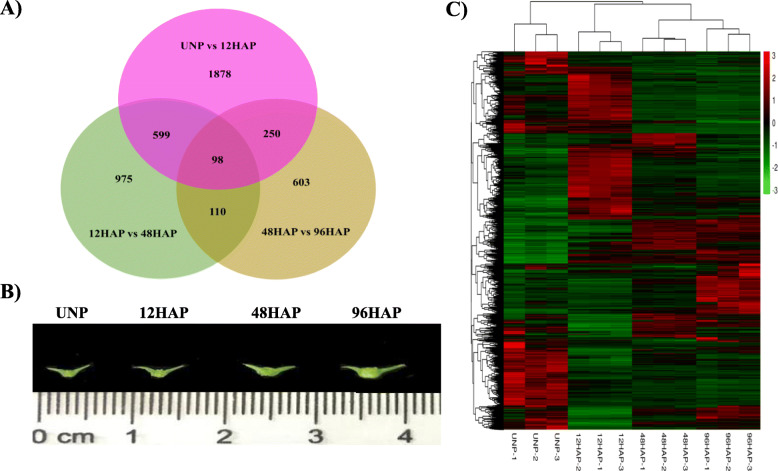
**a** Unique and shared differential expression of unigenes in UNP vs 12HAP, 12 vs 48HAP, and 48 vs 96HAP pairwise analysis. **b** Representatives sepal phenotypes at different stages including Unpollinated flower sepal (UNP), sepal at 12 h after pollination, before fertilization (12HAP), sepal at 48 h after pollination (48HAP), and sepal with early visible symptoms of development at 96 h after pollination (96HAP). **c** Hierarchical heatmap showing the variability among replicates of each treatment for DEGs

To reveal the alternation in gene expression during sepal development after pollination, the non-strand-specific cDNA libraries were constructed from Cornel-9 unpollinated (UNP) and pollinated (12, 48, and 96 HAP) flower sepals with three biological replicates. A total of 148,241,329 paired-end clean reads that were 150 bp in length from 12 libraries were generated. 12 to 14.1 million clean reads per library from unpollinated and 9 to 14.8 million clean reads per library from pollinated flower sepals were generated. Clean reads were mapped to the spinach draft genome [16]. The mapping rate was over 91% for samples of each stage (Table S1). Differential expression analysis was conducted by a continuous comparison system to determine the differentially expressed genes at each stage after pollination. A total of 2825 genes were expressed differentially between unpollinated (UNP) and 12 HAP with 1443 upregulated and 1382 downregulated genes, decreased to 1782 between 12 HAP and 48 HAP with 715 upregulated and 1067 downregulated genes, and 1061 between 48 and 96HAP with 696 upregulated and 366 downregulated genes. The summary of DEGs in all designed comparisons is reported in Fig. 1a. Variability among the replicates of each treatment for DEGs is presented by a hierarchical heatmap in Fig. 1c.

### KEGG and GO enrichment analysis

To further confirm the functional annotation, we performed the KEGG enrichment analysis for differentially expressed genes at each selected time point (Figure S2). The results suggested that the ‘plant phytohormone signal transduction’ was the significantly enriched pathway in all comparisons, while the other pathways such as ‘Tryptophan metabolism’, ‘DNA replication’, ‘Glycin, serine, threonine metabolism’, and ‘Valine, leucine and isoleucine degradation’ pathways were overrepresented at UNP vs 12 HAP. Pathways related to ‘glycan degradation’, ‘carbon metabolism’ at 12 vs 48HAP and ‘Phenylpropanoid biosynthesis’, ‘Flavone and flavonol biosynthesis pathways’ and ‘alpha-Linolenic acid metabolism’ were enriched at 48 vs 96HAP. Additionally, ‘secondary metabolites biosynthesis pathway’ was of significant enrichment at 12 vs 48HAP and 48 vs 96HAP.

To identify up- and down-regulated GO at each selected time point, differentially expressed genes of all pairwise comparisons were subjected to GO enrichment analysis (Figure S3). At the transition from UNP to 12 HAP, ‘cell wall organization’, ‘cell wall modification’, ‘methylation’, ‘cell growth’, ‘developmental growth involved in morphologies’, ‘DNA replication’ were upregulated. However, ‘organ nitrogen compound catabolic processes, ‘oxidation-reduction process’, ‘lipid catabolic process’ were downregulated. At the transition from 12HAP to 48HAP, ‘Carbohydrate metabolism process’, ‘photosynthesis, dark reaction’, reductive pentose-phosphate cycle’ ‘polysaccharide metabolism (glucan)’, ‘electron transport in photosystem I’ were significantly upregulated. Downregulated GO terms were mainly ‘chitin metabolic process, ‘amino sugar metabolic processes’, ‘glucosamine catabolic process’. At the transition from 48HAP to 96HAP, ‘cell wall metabolic and biosynthetic process’, ‘xylan biosynthetic process’, ‘negative regulator of peptidase and hydrolase activity’ was significantly upregulated. The overrepresentation of ‘microtubule-based movement’, ‘cell division’ ‘mitotic cell cycle’, ‘cytokinesis’ was enriched in the downregulated GO group (Figure S3).

### Tryptophan-dependent auxin biosynthesis pathway after pollination

**Fig. 2:**
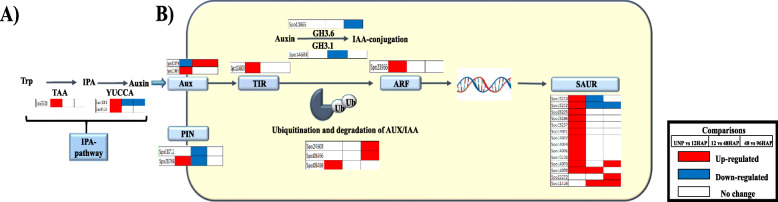
KEGG analysis emphasized the differential gene expression of genes involved in **a** Trp-dependent auxin biosynthesis and **b** auxin signal transduction pathway

In higher plants, auxin is biosynthesized from the tryptophan (Trp) by the indole-3-pyruvic acid (IPA) pathway [17]. It’s a two-step process that involves the amino group removal from Trp forming indole-3-pyruvate (IPA), catalyzed by TRYPTOPHAN AMINOTRANSFERASE OF ARABIDOPSIS (TAA) family and then IPA decarboxylation by YUC flavin mono-oxygenase enzymes (YUC), forming IAA. In this study, 1 TAA (*Spo25321*) and two YUC (*Spo11200, Spo24134*) transcripts were found to be upregulated at UNP vs 12HAP (Fig. 2a, Table S2).

### Phytohormone signal transduction insight reveals enrichment in auxin signaling pathway

KEGG enrichment analysis revealed regulation in phytohormone signal transduction after pollination. A deep insight into this pathway revealed the clearly defined enrichment in auxin transport and signaling pathway (Fig. 2b, Table S2). Auxin is unique among other plant hormones for having polar transport due to its weak acidic nature and its intercellular movement is accomplished by auxin influx AUXIN1/LIKE-AUX1 (AUX/LAX) and auxin efflux carriers PIN-FORMED (PIN) proteins. Three AUX1 transcripts expression found to be accumulated after pollination with two transcripts at UNP vs 12HAP and one at 12 vs 48 and 48 vs 96HAP. Besides that, 2 PIN transcripts downregulated at 12 vs 48 h after pollination suggest further auxin accumulation. Two AUX/IAA (auxin response factors repressors) genes showed non-significant change in expression pattern at UNP vs 12 HAP, which promotes the transcription of ARF (AUXIN RESPONSIVE FACTORS) at 12 HAP. ARF mediated early small auxin up-regulated RNA genes (14 SAUR) were differentially regulated after pollination with upregulation of 12 SAUR genes at UNP vs 12HAP, however, only a few genes were upregulated at 12 vs 48HAP and 48 vs 96HAP. Decrease expression of some Gretchen Hagen 3 (GH3) transcripts such as GH 3.1 and GH3.6 involved in auxin conjugation, suggested that there would also be some active auxin accumulation at later time points. Expression data suggest that significant auxin signaling is induced at 12 HAP to regulate growth responses at later time points.

### Cell expansion and cell division are altered after pollination

Surveying the auxin signal transduction pathway clearly showed a large portion of DEG’s after pollination. Auxin is a major regulator of plant development and growth. Many aspects of these processes involved multiple auxin exerted controls on cell wall expansion and cell division. Further, a significant expansion in cell size was observed after pollination as sepal grows (Fig. 6A). This led us to raise a question about (1) cell expansion, modification, and division gene expression increase after pollination. To address this, we examined expression pattern of genes involved in these pathways. Cell expansion requires cell wall loosening that is primarily regulated by endotransglycosylase/hydrolases (XTHs) and expansins (EXP). Sixteen putative members of the expansin family were differentially expressed in the pollinated sepals. All these genes were up-regulated at UNP vs 12 h after pollination except for 1 gene that has up-regulation at 48 vs 96HAP. Eight genes among 16 expansin genes were downregulated through the 12 to 48HAP and 48 to 96HAP transitions. A total of 8 genes were annotated as XTHs were differentially expressed after pollination, and these genes were up-regulated at 48 vs 96HAP with 4 genes also up-regulated at UNP vs 12HAP. In addition, the modulation of several genes involved in cell wall modification and homogalacturonan breakdown was observed. Twenty-three pectinesterase genes were regulated after pollination. Seventeen of these genes were up-regulated at UNP vs 12HAP and 6 genes at 12 vs 48HAP. Downregulation of 9 and 11 genes was observed at 12 vs 48 and 48 vs 96 h after pollination, respectively. This may indicate that the pectin polymers are broken down rapidly for recycling after pollination. Enrichment of most of the cell wall loosening and pectinesterases may suggest that cell enlargement signals start rapidly right after pollination at 12H and continue at later time points (Table 1).

**Table 1:** Differentially expressed genes involved in cell wall degradation pathway

Gene	UNP vs 12HAP	12HAP vs 48HAP	48HAP vs 96HAP	Description
*Spo03896*	7.46734057	−1.22016	− 1.945425837	Expansin
*Spo03955*	6.53648645	−1.95405	−1.779265134	Expansin
*Spo03956*	6.41057636	−1.89786	−1.585782477	Expansin
*Spo04175*	8.62245491	−2.20915	− 2.672611741	Expansin
*Spo07257*	1.73653856	− 0.01874	− 0.2080243	Expansin
*Spo08184*	1.97650776	− 0.80042	− 0.3022703	Expansin
*Spo09164*	5.38590535	−1.45931	−1.982754	Expansin
*Spo10792*	1.30661261	0.097526	0.06754917	Expansin
*Spo11773*	−1.4006305	0.094508	1.881701	Expansin
*Spo16876*	1.5024521	− 0.26965	− 0.708257636	Expansin
*Spo16879*	1.28083685	−0.05457	1.729898838	Expansin
*Spo19964*	1.44385389	−0.57618	0.1417831	Expansin
*Spo22805*	7.1862212	−1.58435	−2.498659514	Expansin
*Spo24883*	6.90757885	−1.83147	−1.412926735	Expansin
*Spo25442*	5.46642129	−1.92219	−2.686797576	Expansin
*Spo11770*	1.48279765	0.996554	−1.52406816	Expansin
*Spo00609*	−3.297828	1.921533	2.862723211	Xyloglucan endo-transglycosylase
*Spo12328*	−1.5237793	0.422253	2.374772853	Xyloglucan endo-transglycosylase
*Spo10906*	2.67295913	−1.85528	2.110330172	Xyloglucan endo-transglycosylase
*Spo13620*	1.25184116	−0.31193	2.84146497	Xyloglucan endo-transglycosylase
*Spo00611*	0.1074057	0.447591	1.8787089	Xyloglucan endo-transglycosylase
*Spo09254*	0.5353523	0.756239	1.9263372	Xyloglucan endo-transglycosylase
*Spo24498*	1.84504958	0.589429	0.800510154	Xyloglucan endo-transglycosylase
*Spo23065*	1.20312218	−2.08456	1.318063869	Xyloglucan endo-transglycosylase
*Spo07761*	−0.0339172	1.448162	0.692918763	Pectinesterase
*Spo10763*	0.17035098	1.153761	0.933041529	Pectinesterase
*Spo28257*	0.36117586	−1.61298	0.2091781	Pectinesterase
*Spo09269*	0.53544282	1.641543	−0.533757927	Pectinesterase
*Spo08782*	0.58530213	1.94774	0.657921518	Pectinesterase
*Spo04512*	0.61298854	1.416131	0.357255899	Pectinesterase
*Spo27330*	1.0339677	0.317981	−0.468142586	Pectinesterase
*Spo04812*	1.12271049	0.871412	−2.233404127	Pectinesterase
*Spo05630*	1.34702681	1.015853	0.606978822	Pectinesterase
*Spo14737*	1.51883485	−0.14902	0.458649816	Pectinesterase
*Spo06997*	1.5900245	0.706009	0.827795663	Pectinesterase
*Spo17580*	2.01241294	0.613736	−0.4723155	Pectinesterase
*Spo17238*	3.29815995	−1.25997	0.761623481	Pectinesterase
*Spo03151*	5.32045056	−1.46931	−2.192662744	Pectinesterase
*Spo24512*	5.84478345	−1.19687	−2.191944367	Pectinesterase
*Spo18633*	5.85209807	−1.48403	−2.577665667	Pectinesterase
*Spo03538*	6.39078316	−0.76487	−2.182242868	Pectinesterase
*Spo13817*	6.76310559	−1.36563	−1.964692024	Pectinesterase
*Spo17646*	7.0151214	−0.66097	−3.868810018	Pectinesterase
*Spo18632*	7.27261063	−1.4638	−1.776909947	Pectinesterase
*Spo24511*	7.62192398	−0.95735	−2.611992976	Pectinesterase
*Spo18631*	8.33387766	−1.0553	−2.08528221	Pectinesterase
*Spo22192*	8.57263651	−1.56241	−2.589320543	Pectinesterase

**Fig. 3:**
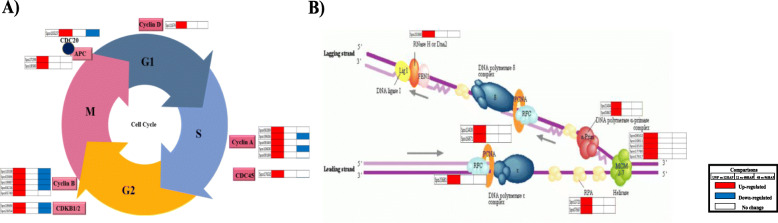
Differential expression of gene involved in cell-cycle. **a** Cell cycle checkpoints. **b** DNA replication

We then surveyed key genes involved in cell division that encompasses the sequence of events [18]. The initiation of active replication (S-phase) requires the assembly of proteins including replication factors RFA, minichromosome maintenance protein complex (MCM), DNA polymerases, proliferating cell nuclear antigen (PCNA), and other factors. Five MCM genes (MCM 2, 3, 4,6,7), two DNA polymerase α primase complex genes (PRI1and POLA2), two PCNA genes, and three replication factor (RPA1, RPA2, and RFC3) were found to be up-regulated at UNP vs 12HAP, while no regulation was observed through12hap to 48hap and 48hap to 96hap transitions (Fig. 3b, Table S2). Cyclins, Cdks, and APC/C involved in cell cycle phase transitions were found to be regulated after pollination. Eleven genes annotated as cyclin, key cell cycle regulators triggering G1 to S and G2 to M transitions [19] were differentially expressed after pollination. Five genes showed high homology to cyclin-A, 5 genes with cyclin B, and 1 gene with cyclin D was upregulated at UNP vs 12HAP. Among these, 2 cyclin A and 4 cyclin B genes were downregulated through 48HAP to 96HAP transition. None of these genes were found to be regulated at 12HAP vs 48HAP. Cyclins regulate the cell cycle events by partnering with an enzyme family called cyclin-dependent kinases (Cdks). Two CDKB genes (CDKB1 and 2) were upregulated only at UNP vs 12HAP while downregulated at 48 vs 96HAP. The Anaphase-promoting complex (APC/C), another cell cycle regulator causes protein degradation that holds sister chromatids and allowing them to move to opposite poles of the cell during anaphase. They also cause M cyclins degradation, allowing the new daughter cells to enter G1 by pushing the cell out of mitosis. Two APC gene homologous to APC8 and 10 were upregulated at UNP vs 12HAP and not regulated through the 12HAP to 48HAP and 48HAP to 96HAP transition (Fig. 3a). Enrichment of replication machinery genes and cell cycle regulators genes indicate that the cell number of sepal organ start increasing after pollination.

### Cell wall metabolism is altered after pollination

**Fig. 4:**
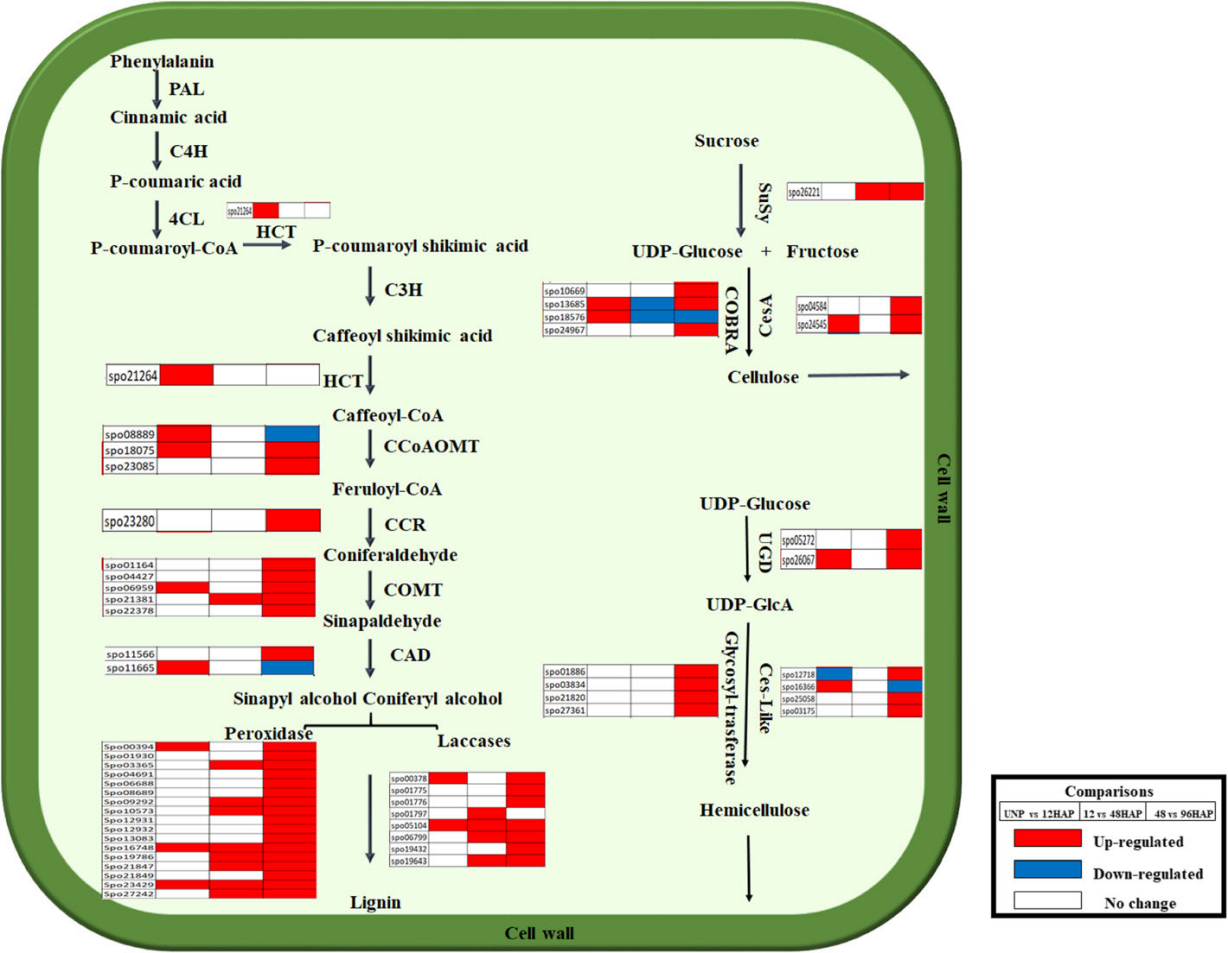
Differential expression of gene involved in cell wall metabolism

A possible direct or indirect role of auxin in cell wall polysaccharides synthesis and the over-representation of cell wall-related genes in GO enrichment analysis led us to study these genes in detail (Fig. 4, Table S2). Cell wall is composed of cellulose, hemicellulose, pectin, and lignin, which cross-link and interact to form a complex and rigid network. Cellulose is the most abundant biopolymer in plant cell wall and composed of UDP-glucose that is catalyzed by cellulose synthase (CS). Two *CESA* genes homologous to *Arabidopsis CESA4* and 8, essential components of the CESA complex in SCWs, were found to be upregulated at 48 vs 96HAP. *COBRA* genes encode GPI-anchored proteins involved in crystalline cellulose assembly during cell wall formation. Three *COBRA-like* genes were predominantly expressed at 48 vs 96HAP, with a similar expression pattern to *CESA* genes indicate that they might cooperatively involve in cellulose assembly and synthesis in cell wall. Hemicellulose is the second important cell wall component, catalyzed by cellulose synthase-like genes and glycosyl-transferases (GT). Four *CSL* genes were found to be differentially expressed after pollination. Among them, 3 genes were upregulated at UNP vs 12HAP and 48 vs 96HAP. In addition, five members of *GT* genes were preferentially expressed at 48 vs 96HAP. Lignin is composed of amorphous polymers monolignols, which are biosynthesized by phenylpropanoid pathway. In total, 12 genes involved in lignin biosynthesis pathway were significantly expressed after pollination. As expected, several lignin biosynthesis transcripts including one *hydroxycinnamoyltransferas*e (HCT), three *caffeoyl CoA 3-O-methyltransferase* (*CCoAOMT*), two *cinnamyl alcohol dehydrogenase* (*CAD*), one *cinnamoyl-CoA reductase* (*CCR*), and five *catechol-O-methyltransferase* (COMT) were differentially expressed after pollination. Finally, the monolignols are polymerized by peroxidases and laccases, then transported to the cell wall. Eight *laccase* (*LAC*) and 17 peroxidase genes were differentially expressed after pollination with most genes were dramatically upregulated at 48 vs 96HAP. Among numerous enzymes, glycosyl hydrolases (GHs) are associated with cell wall polysaccharides degradation and remodeling. Nine GHs genes were found to be regulated after pollination. Among them, 8 genes were upregulated at 48 vs 96HAP, 3 at 12 vs 48HAP, and 2 at 12 vs 48HAP. The expression patterns of these genes were consistent with the substantial lignification and cell wall biosynthesis genes at 96 h after pollination.

### Photosynthesis and chlorophyll contents are altered in pollinated flower sepal

**Fig. 5:**
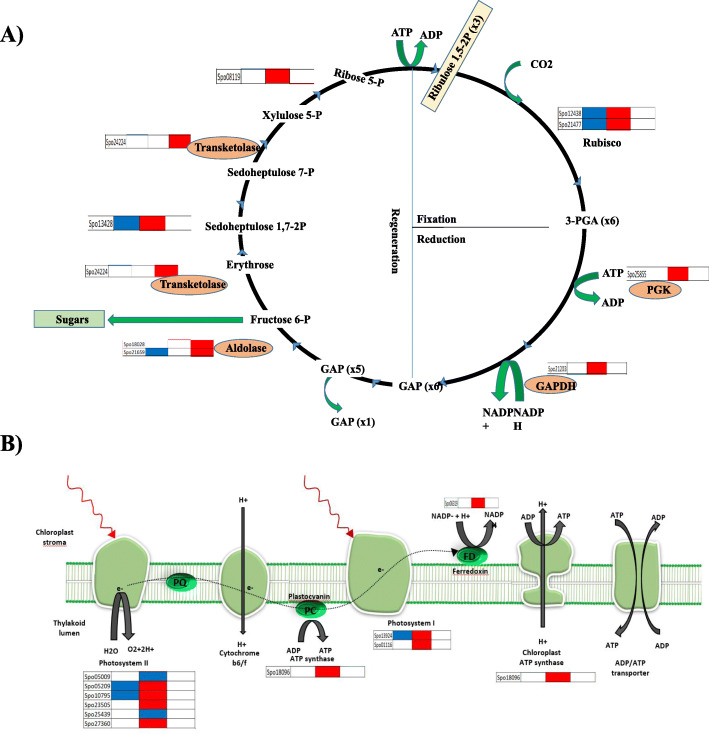
Display of gene expression of genes. **a** Calvin cycle. **b** Photosynthesis light dependent reaction

**Fig. 6:**
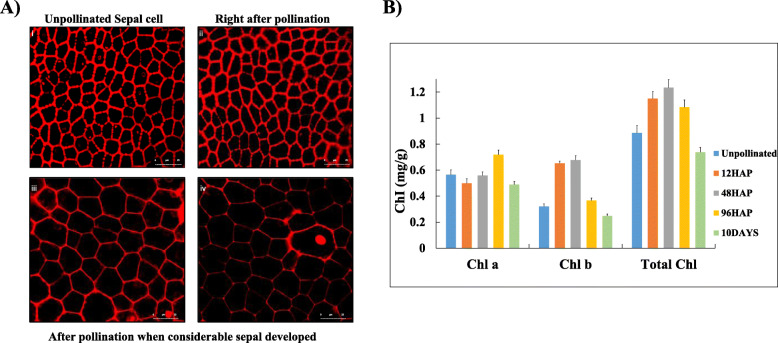
**A. (i)** Unpollinated flower sepal cells **(ii)** Sepal cells right after pollination **(iii & iv)**. Cell expansion can be observed when considerable sepal is developed after pollination. **B.** Chlorophyll contents at different time points after pollination

Survey of cell wall metabolism clearly indicates a large portion of DEGs belongs to cellulose, hemicellulose, monolignol polymerization, cell wall loosening, and degradation after pollination. UDP-glucose is the major building block of cell wall and the intermediate in sucrose biosynthesis pathway. This led us to look at the expression pattern of Calvin cycle genes (Fig. 5a, Table S2). We found dynamic changes in gene expression pattern through the 12 to 48HAP transition in all three phases of the Calvin cycle (fixation, reduction, and regeneration) regulated by specific enzymes. Two rubisco small subunit genes, involved in carbon fixation, one phosphoglycerate kinase, and one glyceraldehyde phosphate dehydrogenase (GAPDH) having important roles in photosynthetic carbon reduction found to be upregulated through 12HAP to 48HAP transitions. However, three genes (aldolases and transkeletoses) involved in regeneration of Ribulose 1,5-bisphosphate step that limits photosynthesis found to be upregulated at 48 vs 96HAP. Since sepal enhanced the Calvin cycle after pollination, it was considered that the light-dependent reaction of photosynthesis also intensifies simultaneously to provide metabolic energy (Fig. 5b). The up-regulation of light-harvesting center proteins (4 genes of photosystem II and 2 genes of photosystem I) together with other photosynthesis components such as the electron transporter ferredoxin and ATP-synthase suggested that the photosynthesis apparatus might be attenuated notably at 12 vs 48HAP. Next, we examined whether there is conversion of sepal photosynthate to hexose after pollination. Sucrose synthase (SuSy) reversible convert sucrose to UDP-glucose which is the substrate for biosynthesis of cellulose and the other nucleotide-sugar precursors required for hemicellulose and pectin. In this study, only one Susy was up-regulated at 12 vs 48 and 48 vs 96HAP. As chlorophyll is widely considered the direct regulator of photosynthetic capacity in plant leaf (Singsaas et al., 2004), we then measured the sepal chlorophyll contents at each time point after pollination (Fig. 6B). The results indicated that total chlorophyll contents increase at 12, 48 HAP when sepal growth just starts, and then slight decrease at 96HAP, but still more than unpolllinated samples. A significant decrease in these contents was observed after 10 days of pollination when sepal has been grown enough to suggest that photosynthetic activity enhances right after pollination to provide precursor for growth and eventually drops when significant growth occurs.

### RNA-seq data validation by qRT-PCR

**Fig. 7:**
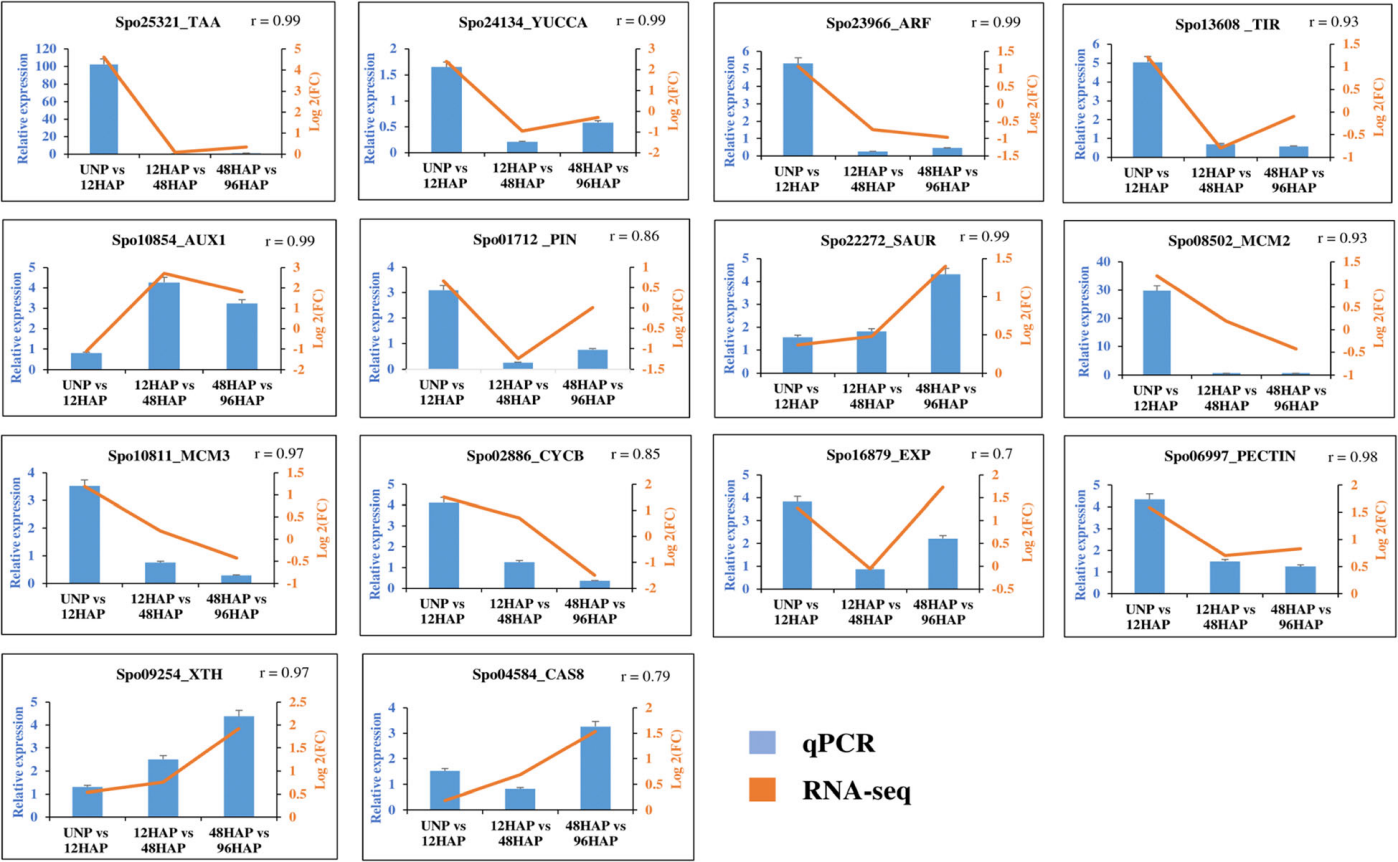
qPCR analysis for expression confirmation, Blue bars represents the relative expression in qPCR, and orange line represents the log2 (FC) values in transcriptome for corresponding genes

To validate expression pattern of genes identified by RNA-seq data, 14 genes were randomly selected and examined by qRT-PCR. Two of the auxin biosynthesis genes (*Spo25321, Spo24134*), three genes from auxin signal transduction pathway (*Spo01712, Spo23966, Spo13608*), three cell cycle genes (*Spo08502, Spo10811, Spo02886*) characterized by higher expression at 12HAP compared to UNP. Quantitative PCR analysis confirmed differences in transcript abundance between cell wall biogenesis genes (*Spo09254, Spo04584*) and SAUR (*Spo22272)* at 48 vs 96HAP, much higher than12 vs 48 HAP and UNP vs 12HAP. Two cell expansion genes (*Spo16879, Spo06997*) and AUX1 (*Spo10854*) genes also showed similar expression patterns in RNA-seq and qPCR. Expression patterns of all selected genes were confirmed to be consistent with the RNA-seq data (Fig. 7).

### Auxin acts as a signal and triggers autonomous spinach sepal development

**Fig. 8:**
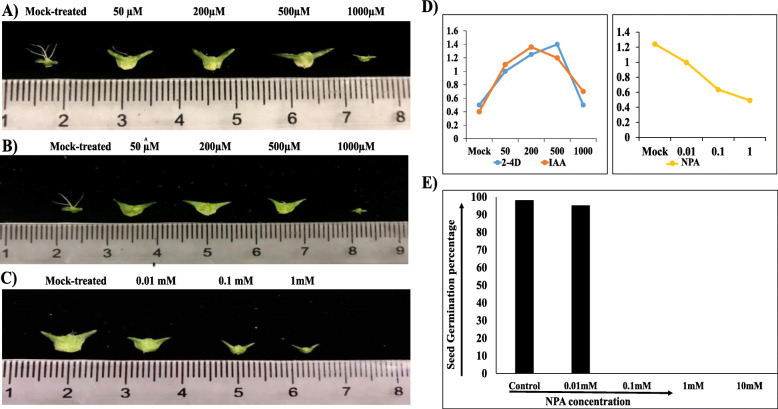
Developmental changes observed after 10-days of auxin and auxin transport inhibitor treatment. **a** IAA treated unpollinated flower’s sepals at mock-, 50, 200, 500, and 1000 μM. **b** 2,4D treated unpollinated flower’s sepals at mock-, 50, 200, 500, and 1000 μM. **c** NPA-treated pollinated flower’s sepals after 12HAP at mock-, 0.01, 0.1, and 1 mM concentration gradient in female spinach variety “Cornel-9”. **d** Quantitative sepal development (cm) under hormone treatments at different concentrations. **e** Germination percentage of seeds obtained from NPA-treated pollinated flowers at maturity

RNA-seq analysis underlined that sepal development initiating after pollination may drive by cell expansion, cell division, and cell wall remodeling. Given that auxin signaling is active in the developing sepal after pollination, and this hormone is known for having a role in cell growth and expansion, we tested whether exogenous application of auxin provides signal to drive sepal development. We treated unpollinated spinach flower with synthetic 2,4-D and natural IAA auxin analog at 50, 200, 500, 1000uM concentration, and investigated autonomous sepal development after 10 days (Fig. 8a). We measured the size of the 15–20 sepals to estimate the average growth. At 50, 200, and 500uM, unpollinated flower sepal develop almost equally, and their growth was comparable with that of pollinated flower sepal but at 1000uM sepal wilted and did not grow at all for both synthetic with a slight increase at natural auxin analog, which indicates that over-accumulation of auxin hormones in the integuments (sepal) of spinach flower would trigger sepal abscission responses. To further confirm the effect of auxin, we treated spinach flower with NPA at 0.01, 0.1, 1 mM concentration at12HAP and investigated autonomous sepal development after 10 days (Fig. 8b). At 0.01 mM, flower sepals normally develop as mock-treated ones slightly smaller in size, but at 0.1 and 1 mM sepal development varies significantly, At 0.1 mM sepals grow a bit but smaller than 0.01 mM and mock-treated ones, at 1 mM sepal did not grow at all. We also observe the difference in seed size in pollinated flowers after NPA treatment at maturation. At 0.01 mM concentration, normal seeds as mock-treated ones, while aborted seeds of small size at 0.1 and 1 mM NPA concentration were developed. We further check the germination percentage of the seeds in each NPA treatment individually (Fig. 8c). A hundred seeds of each treatment were observed for germination and the germination percentage was calculated. In mock-treated ones, the germination percentage was about 97%. In 0.01 mM, about same germination percentage was observed as in mock-treated seeds as their sepal size was also similar, while in 0.1 and 1 mM NPA treatment, no seed germinated and consistent with their sepal size which also not developed at all. This might suggest that auxin transport through an interactive pathway may drive ovule and sepal development in spinach after pollination as sepal grows with the encased developing seeds. The quantitative data of sepal size in each treatment is illustrated in Fig. 8d.

## Discussion

Sepal retention and development after pollination unique to spinach female flower may determine by pollination signals and how is it regulated remains unclear. In this study, expression pattern of DEGs between unpollinated and pollinated flower sepal at 3 different time points with a continuous comparison system (UNP vs 12HAP; 12 vs 48HAP; 48 vs 96HAP) revealed that sepal development after pollination may be related to three biological processes, including (i) response to phytohormones (ii) cell division and expansion (iii) cell wall biogenesis. KEGG enrichment analysis of DEGs suggested that plant phytohormones signal transduction pathway was of significant importance after pollination at each time point. Especially, the auxin-related DEGs in signal transmission process, suggesting that auxin had a much greater influence on the regulation of sepal growth and development. After pollination, the auxin influx carrier AUX1 genes were upregulated, accompanying with increased transcripts level of SAURs- the auxin response genes. And the downregulation of genes participated in auxin conjugation will stimulate more active auxin contents in sepal cells. This was further confirmed by exogenous NPA application through the weakening of auxin flow in pollinated flower sepal which did not show any developmental change at 0.1 and 1 mM which suggested that auxin accumulation is associated with sepal development and weakening of auxin flow disrupt the development.

Pollination is perceived at stigmatic surface, and developmental events are initiated with the pollen-stigma interaction or a pollen-borne substance. Auxin was proposed as a primary pollination signal by direct transfer of pollen-borne auxin or other factors to the stigma [20]. In the style of tobacco flower, strong correlation between auxin synthesis and pollen tube growth was found [21, 22]. Nitsch [23] found that auxin concentration increase on the tryptophan-auxin precursor contacting medium after 13.5 and 20.5 h after germination of pollen. That suggested that pollen tube possessed an enzyme system that converts tryptophan to auxin. It was also found that resting pollens and unpollinated ovaries have a tryptophan-IAA enzyme that’s activity enhanced after pollination in corn [24]. Later in higher plants, Trp-dependent TAA–YUC-mediated auxin biosynthesis was found a major contributor for the auxin pool [25]. In our study 2 YUC and 1 TAA found to be upregulated indicate that Trp-dependent auxin biosynthesis provides pollination signal for sepal retention in spinach. Further, application of auxin analog treatment to unpollinated flower sepal suggests that auxin is sufficient for sepal retention and growth after pollination but the developmental effect is found to be concentration-dependent. We examined that at 50, 200 and 500 uM 2,4 D and IAA treatment, the unpollinated flower sepal develop same as pollinated one but 1000 uM concentration elicited different response (Fig. 8a, b) suggested that over-accumulation of auxin may trigger regular pollination-regulated perianth senescence response. Studies suggested that spraying with auxins is, however, not always successful [26, 27]. At certain concentrations, auxin may enhance rather than lessen abscission, but it is well known that excess auxin stimulates ethylene production [28]. In orchid flowers, auxin-regulated ACC synthase is rapidly induced in the stigma by pollination, resulting in burst of ethylene production leading to flower abscission [29]. In our study, no gene related to ethylene was found to be regulated indicate that pollen-derived auxin synthesis may be insufficient to be the sole primary pollen signal for ethylene synthesis rather it triggers sepal development in spinach. It is also possible that pollen contains other auxin forms, such as auxin conjugates, or additional factors, which may synergistically participate with auxin to evoke regular pollination-regulated perianth abscission responses in spinach.

Auxin control all plant developmental aspects by regulating cell expansion, division, and remodeling. Rapid cell expansion requires wall loosening that is accomplished by modifying the molecular interactions within the cell-wall network and relaxation of wall tension. Several proposed models suggested that loosening of cell wall resulted from breakage of crosslinks, cleavage or weakening of the non-covalent bonds that facilitate sliding of hemicellulose polymers along the cellulose scaffold. Expansins (EXP), xyloglucan endotransglycosylase/hydrolases (XTHs) and pectin methylesterases (PME) have been identified as major cell wall-loosening agents [30–32] found to be upregulated right after pollination in this study. The activity of several EXP and PME family member were induced in response to auxin in roots [33]. Positive role of SAURs in cell expansion has been revealed from several recent studies in Arabidopsis [34–36]. Accumulating evidence also indicates that auxin can directly influence key regulators (cyclin and cyclin-dependent kinase) of cell cycle phase transition to control cell proliferation [37]. Global transcript profiling analysis revealed that *CYCB1* and *CYCA2* were potentially induced by auxin. Cyclin genes were found to have auxin-responsive elements (AuxREs) in the promoter region; however, the functional relevance of such elements has not yet been investigated [38, 39]. In our study, various cyclin A, B together with genes involved in DNA replication like helicases, DNA polymerases, replication factors during the S-phase of cell cycle are also upregulated suggesting that auxin genes expression accumulation after pollination may trigger the cell cycle-related genes in sepal for cell proliferation.

Apart from cell wall expansion and cell proliferation, associations between hormone signaling and cell wall biosynthesis have long been suggested. Cell wall biogenesis also plays a critical role in establishing cell size during development and partially regulated by auxin. The supply of glucose precursors in the cell wall biogenesis could thus be provided by the increased photosynthate production, breakdown of cell wall polymers, or other metabolic pathways [40]. Studies demonstrated that auxin response factors (ARFs) overexpression regulate photosynthesis rate and sugars accumulation by increasing chlorophyll contents in the leaves and fruits of tomato plants [41]. Key genes involved in Calvin cycle for sucrose biosynthesis like rubisco-subunit, PGK, GAPDH, FBP, aldolase, and treskeletose genes were found to be upregulated after pollination. Regulation of cell wall metabolism and increased cell growth following pollination is also demonstrated with higher expression of genes involved in cell wall catabolism and anabolism. The upregulated expression of cellulose synthase, *COBRA*, xylan synthesis, glycosyl transferase, and lignin biosynthetic genes in sepal after pollination provides working basis for future spinach breeding. In addition to growth in cell wall biosynthesis, recycling of cell wall polymers by breaking down existing ones via glycoside hydrolases can conserve precursors (glucose) for cell wall reconstruction. Several changes in primary wall architecture have been associated with auxin, including cell wall acidification [42], de novo polysaccharide synthesis, and modification of specific cell wall polymers [43]. Vascular auxin transporter mutants displayed reduced cell wall thickness by affecting xylary fibers in stems, that could be rescued by exogenous auxin application [44]. In mutants of *CCR1 and C4H*, with reduced lignin accumulation, many auxin response genes were down-regulated [45]. Thiel A Lehman [46] demonstrated that disrupted cell wall biosynthesis perturbs auxin transport impacting both isotropic anisotropy growth that overall affects root morphology.

**Fig. 9:**
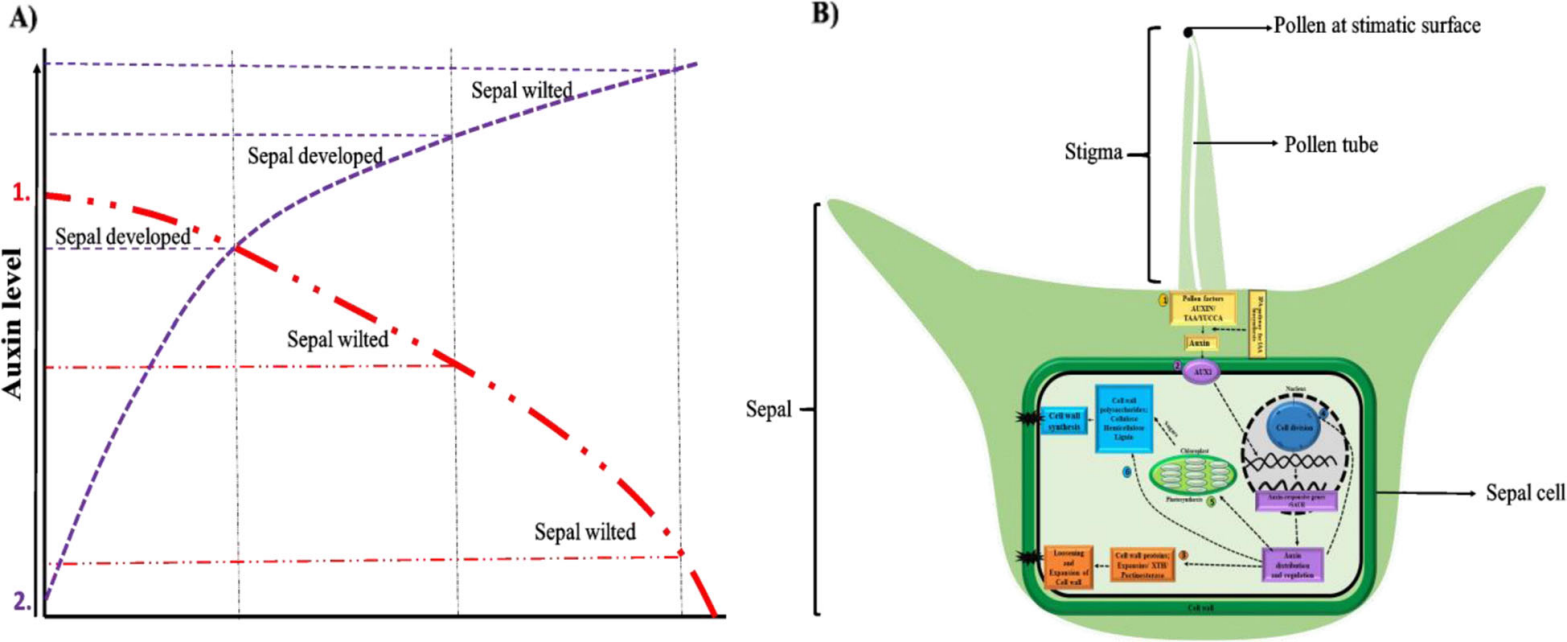
**a** Auxin model shows how auxin concentration affects sepal development in spinach 1. Auxin transport inhibitor (NPA) treatment to pollinated flower (red dotted line) resulted in polar auxin flow weakening that might drop the auxin concentration to level resulted in sepal wilting 2. Increase in auxin concentration under auxin analogs (2, 4D, IAA) application to unpollinated flower (purple dotted line) resulted in sepal development in unpollinated flower sepal at certain concentration, further increase in auxin resulted in sepal wilting indicate auxin level is critical for sepal development. **b** Auxin regulatory metabolic changes for sepal size modulation in spinach. It shows the influence of Trp-dependent auxin biosynthesis and auxin signaling pathway on cell division, expansion, and cell wall biogenesis to modify sepal size in spinach female flower following pollination 1.  Pollination resulted in pollen factor activation which biosynthesizes auxin and 2.  activates auxin signaling pathway 3.  auxin regulate gene responsible for cell wall loosening/expansion 4.  and cell division pathway for sepal cell proliferation for development 5–6. Auxin also  indirectly (through photosynthesis) or  directly regulate cell wall synthesis pathway for biomass accumulation for sepal development

In conclusion, RNA-seq data analysis, 2, 4-D, IAA and NPA treatments demonstrated that auxin played an important role in spinach sepal development following pollination. The results showed that proper auxin concentration and auxin homeostasis were necessary for sepal development (Fig. 9a). Our hypothetical model outlines all the findings in this study and accentuates the influence of Trp-dependent auxin biosynthesis and auxin signal transduction on cell division, expansion, and cell wall biogenesis to modify sepal size in spinach female flower following pollination (Fig. 9b). However, the sepal tissues are pretty small, making it onerous to dissect and collect enough samples acquired for endogenous auxin contents measurement before pollination. Further, this study will serve as an empirical foundation for future molecular studies of the detailed mechanism of sepal retention and development after pollination.

## Methods

### Plant material

Spinach variety Cornel-9 seeds were obtained from USDA. Seeds were treated with 10% hypochlorite solution, incubated for 18 h at 20 °C, and transferred in petri plates on filter paper. After 2 weeks, seedlings at two leaves stage were transferred into 16-cm pots and moved to a greenhouse of Haixia Institute of Science and Technology (HIST), Fujian Agriculture and Forestry University Fuzhou, China with temperatures set at 23 °C, humidity 65%, and a 16 h photoperiod. At the onset of flowering, female and male plants were covered separately to prevent random pollination.

### Pollen tube growth measurements

After flowers get mature, female flowers were pollinated. Ten flowers were collected at 0, 10, 12, 14 h after pollination (HAP) and submerged in 1:9 ratio of Acetic acid and 100% ethanol to fix the samples for 2 h followed by submersion in 1 M NaOH for 6 h at room temperature. Finally, the flowers were washed with 50 mM PBS solution and stained with 0.1% aniline blue for 30 mins. Flowers were fixed on slides and observed under an inverted epifluorescence Leica DM IRB microscope (Wetzlar, Germany) equipped with a Q Imaging Retiga 2000 cooled digital camera (Burnaby, BC, Canada).

### Selection of stages, RNA isolation, library preparation, and high-throughput sequencing

To investigate genes for post-pollination sepal development, flowers were pollinated. Three stages of pollinated flowers were chosen (Fig. 1b). Unpollinated (control; sepal size of about 0.4 cm) and three stages of pollinated flowers were harvested at 12hap (1st stage: before fertilization, when pollen tube about to reach to the ovule, with sepal size of about 0.4 cm), 48hap (2nd stage; early developmental symptoms when sepal just start to develop; sepal size of about 0.55 cm) and 96hap (3rd stage; visible sepal development; sepal size of about 0.75 cm) (Fig. 1b). Sepals were collected in the middle of the stem and size of the sepals was estimated from the average size of 10–15 sepals. Three biological replicates at each time point were collected with each replication of 5–20 flowers. Sepal tissues were separated from the flower by anatomic dissection under a microscope and were rinsed with sterile dH_2_O to remove any pollen residues. QIAGEN, RNeasy Plant Mini Kit (QIAGEN, http://www.qiagen.com/) was used for total RNA extraction, and RNA-seq libraries were constructed using Ultra RNA Library Prep Kit (NEB, #E7770L) according to the manufacturer’s instructions. Illumina Hiseq™2500 system was used to perform high-throughput sequencing of indexed libraries to obtain 150-nt pair-end reads.

### Differentially expressed genes analysis, metabolic pathways, and gene ontology enrichment analysis

Following sequencing, raw reads were checked for quality (adapter sequences, low-quality reads, and other contaminants) via FastQC application https://www.bioinformatics.babraham.ac.uk/projects/fastqc/. Adapter sequences were trimmed out by the Trimmomatic (Version 0.4.4). After that clean reads were mapped to the spinach genome (http://www.spinachbase.org/) and read counts were generated with STAR aligner [47]. Mapped reads referring to each transcript were assembled and FPKM values were calculated using StringTie. For differential gene expression analysis, read count data was normalized and differentially expressed genes (DEGs) were calculated with the DESeq2 in bioconductor (R) package with parameters log2FC > 1 or < − 1 and *p*-value threshold to 0.05, and enabling independent filtering. The differentially expressed genes were investigated in a continuous comparison system (using each successive stage as the reference point). KEGG pathways and Gene ontology enrichment analysis were performed by an online tool “Omicshare” (https://www.omicshare.com/).

### Quantitative RT-PCR analysis for gene expression

The first-strand cDNA from 1 μg of total RNA was synthesized by using PrimeScript™ RT Reagent Kit with gDNA Eraser. The cDNA synthesis reaction was diluted to a final volume of 80 μl. Quantitative RT-PCR was carried out using the TB Green™ Premix Ex Taq™ II kit (TaKaRa), in a CFX- 96-well Real-Time System (BioRad, USA). The volume of final reaction was 20 μl; 1 μl of cDNA, 1 μm of each primer and10μl of TB Green™ PCR master mix. Amplification program was 95 °C for 3 min; 40 cycles at 95 °C for 10 s and 50 °C for 30 s followed by disassociation stage as instructed by the user’s manual. Each sample was repeated three times. The relative expression of gene was normalized by reference gene *GAPDH* in each sample [48]. Normalization of data was done by 2 -△△ct method Primers used in this study are provided in Table S3.

### Measurement of sepal chlorophyll contents and sepal cell size

Sepals were cleaned to remove other contaminants, and three replicates with 15–20 flowers sepals at, UNP, 12,48, 96HAP were submerged in absolute ethanol solution to dissolve the pigments. After the absorbance values of the solution at different wavelengths (470, 649, and 665 nm) were measured by spectrophotometer (L3, INESA, China). Finally, the chlorophyll a, chlorophyll b, and total chlorophyll contents were calculated according to equations mentioned [49], i. Chl a = (13.95A665–6.88A649)*0.001*1/g ii. Chl b = (24.96A649–7.32A665)*0.001*1/g iii. Total Chl = Chl a + Chl b.

For cell size measurement, sepals from female flowers were dissected at three stages (i. Unpollinated, ii. Right after pollination and, iii. When considerable sepal develop) and treated with Propidium Iodide Ready Flow™ Reagent as instructed by user’s manual. The image was taken by a confocal laser scanning microscope.

### Exogenous application of auxin analog and auxin transport inhibitor on spinach flower

Spinach plants were grown in the same conditions as described previously. All the chemicals used were ordered from https://dotscientific.com. After female inflorescences get mature, unpollinated flowers were dipped into synthetic auxin analogs (2,4-Dichlorophenoxyacetic acid; 2, 4-D) and natural auxin analog (Indole-3-acetic acid; IAA) at the concentration of 50 μM, 200 μM, 500 μM, and 1000 μM respectively. For efficient adsorption, flowers were dipped into the solutions for about 1 min. To counter confirm the effect of these hormones, auxin transport inhibitor (N-1-Naphthylphthalamic acid; NPA) sprayed to pollinated flowers at 12 HAP. The concentrations of NPA were set to 0.0 l mM, 0.1 mM, and 1 mM respectively. To enhance the application efficiency of these hormones and their transport inhibitors, another application was done after 2 days. For each treatment, four biological replications were used. Samples were harvested and observed after 10 days.

## Supplementary Information

**Additional file 1: Table S1.** Statistics summary of different samples. **Table S2.** Log2FC of differentially expressed genes involved in different pathways. **Table S3.** Primes sequences used in qRT-PCR. **Figure S1.** A) Spinach sex types B) Sepal protected seed phenotype C) Characterization of pollen tube growth at 12 h after pollination in ‘Cornel-9’ female spinach flower. **Figure S2.** KO enrichment analysis DEGs in pairwise analysis, values in parentheses () shows q-value of each KO term. **Figure S3.** GO enrichment analysis of upregulated and down regulated DEGs in pairwise analysis, values in parentheses () shows q-value of each GO term.

## Data Availability

The datasets used during the current study are publicly available on NCBI (http://www.ncbi.nlm.nih.gov/bioproject/716151) under the accession number (BioProject: PRJNA716151, SRA: SUB9312724).
