# Supplementary material for: Auxin regulated metabolic changes underlying sepal retention and development after pollination in spinach
Source: BMC Plant Biol. 2021 Apr 6;21:166. doi: 10.1186/s12870-021-02944-4 (PMC8022616; doi:10.1186/s12870-021-02944-4)
Supplement: Supplementary file 1 — Additional file 1: Table S1. Statistics summary of different samples. Table S2. Log2FC of differentially expressed genes involved in different pathways. Table S3. Primes sequences used in qRT-PCR. Figure S1. A) Spinach sex types B) Sepal protected seed phenotype C) Characterization of pollen tube growth at 12 h after pollination in ‘Cornel-9’ female spinach flower. Figure S2. KO enrichment analysis DEGs in pairwise analysis, values in parentheses () shows q-value of each KO term. Figure S3. GO enrichment analysis of upregulated and down regulated DEGs in pairwise analysis, values in parentheses () shows q-value of each GO term. [file 12870_2021_2944_MOESM1_ESM.docx]

| **Table S1: Statistics summary of different samples** | | | | |  |  |
| --- | --- | --- | --- | --- | --- | --- |
| **Treatment** | **Time points** | **Source** | **Replications** | **Clean Reads** | **Uniquely mapped reads** | **Uniquely mapped reads %** |
| **Unpollinated** | **UNP** | **Sepal** | R1 | 12027459 | 11158618 | 92.78% |
|  |  |  | R2 | 13293032 | 12285186 | 92.42% |
|  |  |  | R3 | 14130563 | 13161624 | 93.14% |
|  |  |  |  | | | |
| **Pollinated** | **12HAP** |  | R1 | 13716671 | 12794592 | 93.28% |
|  |  |  | R2 | 10493054 | 9687755 | 92.33% |
|  |  |  | R3 | 13658972 | 12488156 | 91.43% |
|  | **48HAP** |  | R1 | 10353470 | 9624230 | 92.96% |
|  |  |  | R2 | 12027453 | 11258959 | 93.61% |
|  |  |  | R3 | 9093643 | 8485895 | 93.32% |
|  | **96HAP** |  | R1 | 12381297 | 11417664 | 92.22% |
|  |  |  | R2 | 14848031 | 13839120 | 93.21% |
|  |  |  | R3 | 12217684 | 11390154 | 93.23% |

**Table S2: Log2FC of differentially expressed genes involved in different pathways**

| **Gene** | **UNP vs 12HAP** | **48HAP vs 96HAP** | **48HAP vs 96HAP** | **Description** |
| --- | --- | --- | --- | --- |
| **Auxin signaling pathway** | | | | |
| Spo23966 | 1.06621 | -0.7415 | -0.962 | ARF |
| Spo03810 | 1.03439 | 0.71938 | 0.2552 | AUX1 |
| Spo10854 | -1.1079 | 2.72404 | 1.81411 | AUX1 |
| Spo21065 | 1.21264 | 0.00923 | -0.4121 | AUX1 |
| Spo13608 | 1.21157 | -0.7976 | -0.0926 | TIR |
| Spo24603 | -0.1791 | 0.29797 | 1.16559 | AUX/IAA |
| Spo08496 | 0.73213 | -0.4331 | 1.86234 | AUX/IAA |
| Spo08498 | 2.00968 | -0.4613 | 0.69513 | AUX/IAA |
| Spo10865 | 0.30231 | -0.7915 | -1.4064 | GH3.6 |
| Spo14689 | -0.1196 | -2.1481 | -0.0444 | GH3.1 |
| Spo15253 | 3.57149 | -1.5893 | -0.1246 | SAUR |
| Spo15252 | 3.1953 | -1.5534 | -1.6049 | SAUR |
| Spo28295 | 1.08762 | 0.47235 | 0.54216 | SAUR |
| Spo15246 | 1.02666 | 0.38932 | -0.1532 | SAUR |
| Spo15237 | 1.19461 | -0.5458 | -0.712 | SAUR |
| Spo14071 | 2.17774 | 0.10196 | 0.94575 | SAUR |
| Spo14072 | 2.03206 | 0.52672 | 0.52053 | SAUR |
| Spo14074 | 2.12944 | -0.6035 | -0.1283 | SAUR |
| Spo14076 | 1.19654 | 0.71934 | 0.9229 | SAUR |
| Spo15250 | 3.01597 | -0.8699 | 0.03258 | SAUR |
| Spo14073 | 2.73616 | -0.3139 | 1.12402 | SAUR |
| Spo14078 | 2.73828 | 1.12093 | 0.8745 | SAUR |
| Spo22272 | 0.36605 | 0.47494 | 1.39813 | SAUR |
| Spo11416 | -0.5699 | 1.78467 | 1.3167 | SAUR |
| Spo01712 | 0.66553 | -1.2422 | 0.00805 | PIN |
| Spo21788 | 1.37288 | -1.0115 | -0.3689 | PIN |
| **Auxin biosynthesis pathway** | | | | |
| Spo11200 | 6.58546 | -1.3133 | -1.9898 | YUCCA |
| Spo24134 | 2.39551 | -0.9481 | -0.2957 | YUCCA |
| Spo25321 | 4.6159 | 0.09248 | 0.33496 | TAA1 |
| **Cell division pathway** | | | | |
| Spo09289 | 2.52754 | 0.36605 | -0.096 | CyclinA-like |
| Spo19636 | 1.27546 | 0.50211 | -1.4229 | CyclinA1 |
| Spo09349 | 1.21488 | 0.58216 | -0.7329 | CyclinA2 |
| Spo10600 | 1.16129 | 0.67692 | -1.4386 | CyclinA2 |
| Spo09189 | 1.94835 | 0.10648 | -0.1909 | CyclinA3 |
| Spo12028 | 1.0415 | 0.71755 | -1.0636 | CyclinB |
| Spo02886 | 1.50577 | 0.7027 | -1.4916 | CyclinB |
| Spo19887 | 1.52059 | 0.68776 | -1.3628 | CyclinB |
| Spo04216 | 1.86711 | 0.72196 | -1.0156 | CyclinB |
| Spo05740 | 1.05267 | 0.85313 | -0.7492 | CyclinB |
| Spo11674 | 1.69372 | -0.2042 | -0.0022 | CyclinD5 |
| Spo17632 | 1.25492 | 0.96751 | -0.1412 | CDC45 |
| Spo10327 | 1.08103 | 0.4057 | -1.1387 | CDC20 |
| Spo19684 | 1.09486 | 0.8166 | -1.0152 | CDKB1 |
| Spo23054 | 1.43441 | 0.60862 | -1.4439 | CDKB2 |
| Spo27299 | 1.19153 | 0.32234 | -0.3694 | APC8 |
| Spo18580 | 1.08074 | 0.64759 | -0.6122 | APC10 |
| **DNA replication** | | | | |
| Spo08502 | 1.18826 | 0.18462 | -0.4312 | MCM2; DNA replication licensing factor MCM2 |
| Spo10811 | 1.34516 | 0.31589 | -0.2397 | MCM3; DNA replication licensing factor MCM3 |
| Spo22535 | 1.5208 | 0.12475 | -0.3136 | MCM4, CDC54; DNA replication licensing factor MCM4 |
| Spo17780 | 1.09636 | 0.34695 | -0.5819 | MCM6; DNA replication licensing factor MCM6 |
| Spo17617 | 1.49409 | 0.0428 | -0.2447 | MCM7, CDC47; DNA replication licensing factor MCM7 |
| Spo21404 | 1.82836 | 0.01882 | -0.2795 | PRI1; DNA primase small subunit |
| Spo05867 | 1.23584 | 0.22633 | -0.1154 | POLA2; DNA polymerase alpha subunit B |
| Spo13439 | 2.3241 | -0.2481 | -0.4612 | PCNA; proliferating cell nuclear antigen |
| Spo16871 | 1.02225 | -0.0119 | -0.1981 | PCNA; proliferating cell nuclear antigen |
| Spo13723 | 1.84462 | 0.35865 | -0.359 | RFA1, RPA1, rpa; replication factor A1 |
| Spo07667 | 1.92786 | 0.01589 | 0.02251 | RFA2, RPA2; replication factor A2 |
| Spo23681 | 1.32942 | 0.22574 | -0.3898 | RFC3_5; replication factor C subunit 3/5 |
| Spo03484 | 1.09482 | 0.25906 | -0.4622 | DNA2; DNA replication ATP-dependent helicase Dna2 |
| Spo23360 | 1.3404 | -0.3257 | -0.2148 | RNASEH2C; ribonuclease H2 subunit C |
| **Photosynthesis pathway** | | | | |
| Spo13924 | -1.1146 | 1.5302 | -0.129 | photosystem I LHC-I |
| Spo01116 | -0.7509 | 1.17829 | 0.28751 | photosystemI polypeptide subunits |
| Spo18096 | -0.591 | 1.14771 | 0.14012 | ATP synthase |
| Spo06319 | -0.8317 | 1.14997 | 0.30829 | Ferredoxin reductase |
| Spo05009 | 0.10252 | -1.242 | -0.3333 | photosystem II LHC-II |
| Spo05209 | -1.4948 | 1.68881 | -0.2533 | photosystem II polypeptide subunits |
| Spo10795 | -1.2956 | 1.14536 | -0.2779 | photosystem II polypeptide subunits |
| Spo23505 | -0.3524 | 1.23626 | -0.002 | photosystem II polypeptide subunits |
| Spo25439 | 0.28917 | -2.241 | -0.0758 | photosystem II polypeptide subunits |
| Spo27360 | -0.8034 | 1.15032 | -0.0345 | photosystem II polypeptide subunits |
| Spo12438 | -1.071 | 1.00889 | 0.67061 | Rubisco small subunit |
| Spo21477 | -1.2162 | 1.98484 | -0.0466 | Rubisco small subunit |
| Spo25855 | -0.2139 | 1.1191 | 0.3357 | Phosphoglycerate kinase |
| Spo21203 | -0.6248 | 1.04299 | 0.03256 | Glyceraldehyde phosphate dehydrogenase (GAPDH) |
| Spo13428 | -1.4589 | 1.32501 | 0.39042 | Seduheptulose bisphosphatase |
| Spo08119 | -0.7176 | 1.04928 | 0.2409 | Ribulose-phosphate 3-epimerase-like |
| Spo18028 | -0.0249 | -0.4126 | 1.19418 | Aldolase |
| Spo21659 | -2.2003 | 0.99107 | 1.15279 | Aldolase |
| Spo24224 | 0.86802 | -0.1467 | 1.01948 | Transketolase |
| Spo26221 | 0.77784 | 1.01262 | 2.0521 | Sucrose synthase |
| Spo05272 | 0.83423 | 0.52796 | 1.37394 | UDP-glucose/GDP-mannose dehydrogenase |
| Spo26067 | 1.69858 | 0.41121 | 0.95769 | UDP-glucose/GDP-mannose dehydrogenase |
| **Cell wall metabolism** | | | | |
| Spo04584 | 0.1862 | 0.6878 | 1.53474 | Cellulose synthase8 |
| Spo24545 | 1.11518 | 0.31031 | 1.47774 | Cellulose synthase2 |
| Spo12718 | -1.3245 | -0.5584 | 1.90601 | Cellulose synthase-like |
| Spo16366 | 1.31767 | 0.85151 | -1.27 | Cellulose synthase-like |
| Spo25058 | 0.37632 | 0.43542 | 1.36617 | Cellulose synthase-like |
| Spo03175 | #N/A | #N/A | 5.58515 | Cellulose synthase-like |
| Spo10669 | -0.0852 | -0.3106 | 1.98123 | COBRA |
| Spo13685 | 3.10175 | -1.4925 | 5.75483 | COBRA |
| Spo18576 | 2.76651 | -1.453 | -1.4107 | COBRA |
| Spo24967 | 0.39294 | 0.50053 | 1.42633 | COBRA |
| Spo01886 | 0.42822 | 0.03902 | 2.94308 | Glycosyl transferase |
| Spo03834 | 0.87931 | 0.3892 | 1.03111 | Glycosyl transferase |
| Spo21820 | 0.60309 | 0.24878 | 1.16902 | Glycosyl transferase |
| Spo27361 | 0.62016 | 0.53783 | 1.80395 | Glycosyl transferase |
| Spo02548 | -3.1036 | -1.7901 | 4.00121 | UDP-glucuronosyl/UDP-glucosyltransferase |
| Spo11151 | -0.324 | -1.8172 | 1.26298 | UDP-glucuronosyl/UDP-glucosyltransferase |
| Spo03349 | -2.2313 | 0.49761 | -1.2744 | Hydroxycinnamoyltransferase (HCT) |
| Spo21264 | 1.05831 | -0.1396 | 0.12083 | Hydroxycinnamoyltransferase (HCT) |
| Spo23280 | -0.2219 | -1.3229 | 1.94159 | Cinnamoyl-CoA reductase (CCR1) |
| Spo08889 | 2.39655 | 0.44029 | -6.9279 | Caffeoyl CoA 3-O-methyltransferase (CCoAOMT) |
| Spo18075 | 1.53056 | 0.42893 | 0.77781 | Caffeoyl CoA 3-O-methyltransferase (CCoAOMT) |
| Spo23085 | -0.1387 | 0.40241 | 1.02882 | Caffeoyl CoA 3-O-methyltransferase (CCoAOMT) |
| Spo01164 | 0.49597 | 0.55373 | 1.01981 | Catechol-O-methyltransferase (COMT) |
| Spo04427 | 0.62705 | -0.7479 | 3.17198 | Catechol-O-methyltransferase (COMT) |
| Spo06959 | 1.11031 | 0.74822 | 2.76708 | Catechol-O-methyltransferase (COMT) |
| Spo21381 | 0.2521 | 1.33832 | 1.0306 | Catechol-O-methyltransferase (COMT) |
| Spo22378 | 0.74908 | 0.47207 | 1.2015 | Catechol-O-methyltransferase (COMT) |
| Spo11566 | 0.62938 | -0.4498 | 2.97568 | Cinnamyl alcohol dehydrogenase (CAD) |
| Spo11665 | 1.10756 | -0.148 | -1.3081 | Cinnamyl alcohol dehydrogenase (CAD) |
| Spo00609 | -3.2978 | 1.92153 | 2.86272 | Glycoside hydrolase |
| Spo02176 | -0.2437 | -0.9013 | 2.02827 | Glycoside hydrolase |
| Spo10906 | 2.67296 | -1.8553 | 2.11033 | Glycoside hydrolase |
| Spo11219 | -5.3698 | -0.4553 | 1.25443 | Glycoside hydrolase |
| Spo13620 | 1.25184 | -0.3119 | 2.84147 | Glycoside hydrolase |
| Spo19813 | 6.28129 | -1.38 | -1.948 | Glycoside hydrolase |
| Spo25143 | 0.48148 | -0.0847 | 2.45297 | Glycoside hydrolase |
| Spo27272 | 0.15721 | 1.56556 | 1.134 | Glycoside hydrolase |
| Spo27307 | 0.91968 | 0.73412 | 1.17958 | Glycoside hydrolase |
| Spo00394 | 1.09082 | 0.51239 | 1.00161 | Peroxidase |
| Spo01930 | -0.1076 | 0.14855 | 1.52699 | Peroxidase |
| Spo03365 | -0.2954 | 1.32623 | 1.69227 | Peroxidase |
| Spo04691 | -0.4742 | 0.12443 | 2.41317 | Peroxidase |
| Spo06688 | -0.2663 | 0.84665 | 1.85994 | Peroxidase |
| Spo08689 | 0.25337 | 0.91237 | 3.88168 | Peroxidase |
| Spo09292 | -2.206 | 1.15854 | 4.10593 | Peroxidase |
| Spo10573 | -1.8292 | 4.04847 | 3.43936 | Peroxidase |
| Spo12931 | 0.2904 | 0.62826 | 1.17344 | Peroxidase |
| Spo12932 | 0.13016 | 0.45196 | 1.12956 | Peroxidase |
| Spo13083 | -0.3296 | 0.90397 | 1.81185 | Peroxidase |
| Spo16748 | 3.00584 | 2.15628 | 2.59453 | Peroxidase |
| Spo19786 | -1.978 | 2.21137 | 1.83169 | Peroxidase |
| Spo21847 | -0.7018 | 1.35549 | 1.81898 | Peroxidase |
| Spo21849 | 0.07221 | 0.87797 | 1.56472 | Peroxidase |
| Spo23429 | 2.38424 | 2.21056 | 3.51293 | Peroxidase |
| Spo27242 | -1.0731 | 1.29461 | 1.58457 | Peroxidase |
| Spo00378 | 1.02214 | -0.7548 | 3.79956 | Laccase |
| Spo01775 | 0.89018 | 0.08809 | 1.0262 | Laccase |
| Spo01776 | -0.0658 | 0.43041 | 1.0184 | Laccase |
| Spo01797 | -0.0334 | 1.07299 | 0.54722 | Laccase |
| Spo05104 | 1.80637 | 3.49244 | 5.40552 | Laccase |
| Spo06799 | 0.47178 | 1.01283 | 1.01782 | Laccase |
| Spo19432 | 0.13572 | 0.45599 | 1.03772 | Laccase |
| Spo19643 | -0.0386 | 1.16744 | 1.03195 | Laccase |
| Spo01446 | 0.0809 | -1.7321 | -1.2285 | Flavonols |
| Spo13035 | -0.7869 | 0.03597 | 2.16606 | Flavonols |
| Spo08585 | -0.5197 | 0.82257 | 1.29662 | Flavonols |
| Spo19296 | -0.2012 | 0.22864 | 1.1294 | Flavonols |
| Spo19590 | 1.03535 | -1.1826 | 2.45049 | Flavonol 3-O-glycosyltransferase |
| Spo21237 | -1.1641 | 1.02578 | 1.36074 | Flavonol 3-O-glycosyltransferase |
| Spo24490 | -3.2017 | -2.9527 | 4.08571 | Flavonol 3-O-glycosyltransferase |
| Spo02331 | -3.6051 | -0.5742 | 4.89957 | Dihydroflavonols |
| Spo10588 | 0.1901 | 0.82613 | 1.35992 | Dihydroflavonols |
| Spo14771 | -0.5721 | 0.26171 | 1.49537 | Dihydroflavonols |
| Spo18990 | -1.4208 | -1.4673 | 3.77092 | Dihydroflavonols |
| Spo25984 | -2.6732 | 2.7999 | 1.85692 | Dihydroflavonols |
| Spo05258 | 0.51051 | 0.96104 | 1.20811 | Dihydroflavonols |

**Table S3. Primes sequences used in qRT-PCR**

| **Genes** | **Primers（5'to3'）** |
| --- | --- |
| Spo25321_TAA_F | GCTACCAAGAAGCATTAGAGGAAGA |
| Spo25321_TAA_R | GTAAGCAAGGTCACATATCACCAA |
|  |  |
| Spo24134_YUCCA_F | TGGTGGCGAATCTTACCTTGG |
| Spo24134_YUCCA-R | TCCTTCCATCACCTTAATCTTCCC |
|  |  |
| Spo23966_ARF_F | ATAGATGATGCTGCGAACAGC |
| Spo23966_ARF_R | ATGCTTGGTATTGCTGATTCTG |
|  |  |
| Spo13608_TIR_F | AGACTTCAGAGGCTTCAGTTC |
| Spo13608_TIR_R | TCAAGACTAGTATACCGGGCT |
|  |  |
| Spo10854_AUX1_F | TCACTGTGGAAATAATGCATGCG |
| Spo10854_AUX1_R | AGCAGTGTCTCTATAAGCAGTC |
|  |  |
| Spo01712_PIN_F | TGCTACATTGTTGCCACATGT |
| Spo01712_PIN_R | CATTGTAGCTTCAGGCAGTCG |
|  |  |
| Spo22272_SAUR_F | ATGAACTTCCACCTCCACCTC |
| Spo22272_SAUR_R | TCTAGTTTCCCTTCTCCTTCATGC |
|  |  |
| Spo08502_MCM2_F | ACACCAACACCAGCAACTTC |
| Spo08502_MCM2_R | TCTTCCTCCTCCTCAACATCC |
|  |  |
| Spo10811_MCM3_F | TCTCCGACCTTCACGACTAC |
| Spo10811_MCM3_R | TCCAACCACAACTTGCTCTC |
|  |  |
| Spo02886_CYCB_F | TCAGGGAGTGGTGGGAAAG |
| Spo02886_CYCB_R | ACCAGCTGATCTATCATTATGAGG |
|  |  |
| Spo16879_EXP_F | TATTCGCGGACTTTGGTCATG |
| Spo16879_EXP_R | TCGTAACATGACCCGCAAC |
|  |  |
| Spo06997_PECTIN_F | AGAGGTAGTGGCATTAGTAGC |
| Spo06997_PECTIN_R | TGACAACAAGGTGAAGATGGTG |
|  |  |
| Spo09254_XTH_F | TCAGCAGGAACTGTTACTGC |
| Spo09254_XTH_R | AGCAGAAGGATCAAACCATAGAT |
|  |  |
| Spo04584_CSA8_F | TCCACCAGAACAACAGATGG |
| Spo04584_CSA8_R | AGAATTATGAGCCGCACAATG |

**
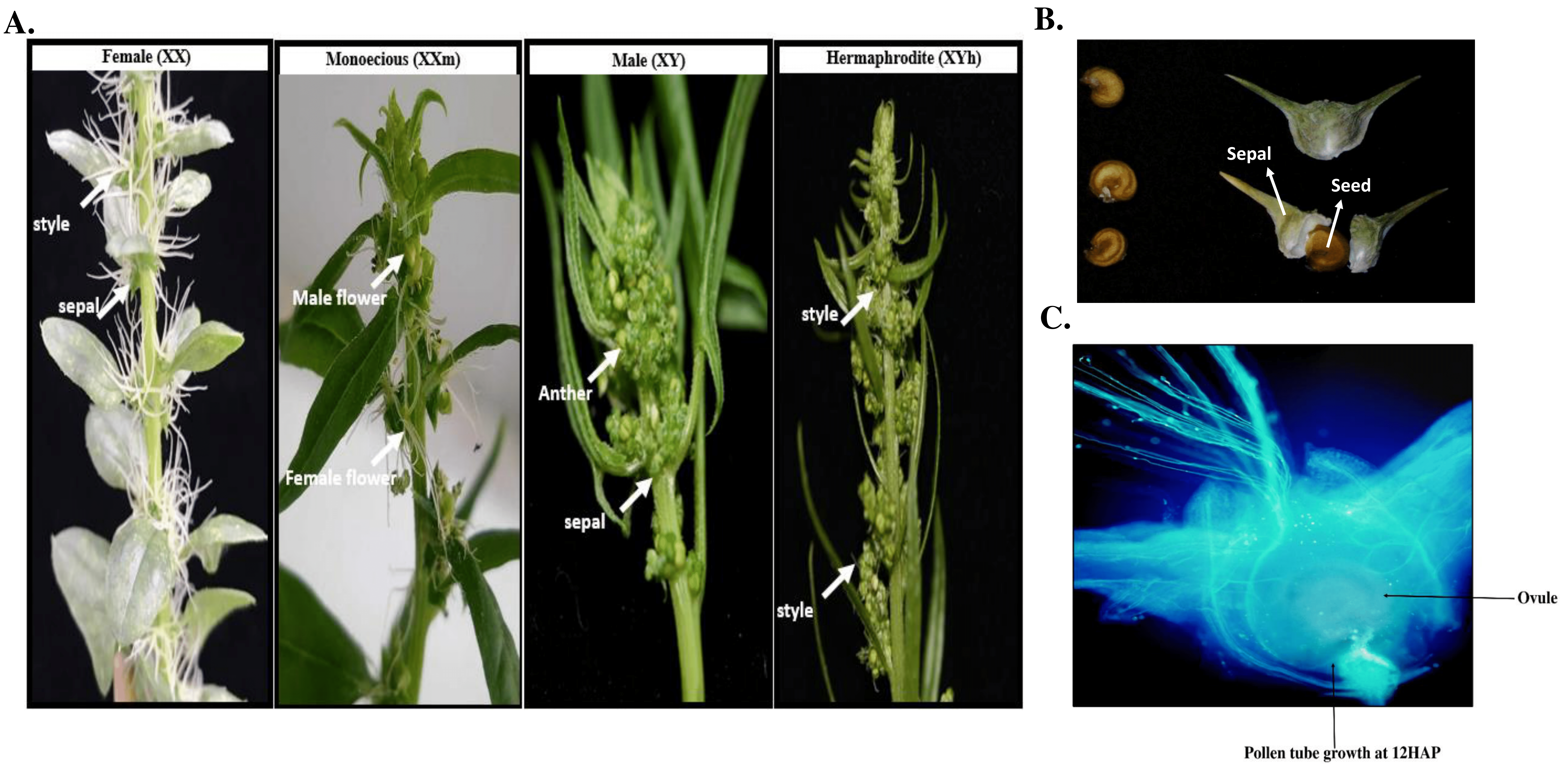
**

**Figure S1: A)** Spinach sex types **B)** Sepal protected seed phenotype **C)** Characterization of pollen tube growth at 12 hours after pollination in ‘Cornel-9’ female spinach flower


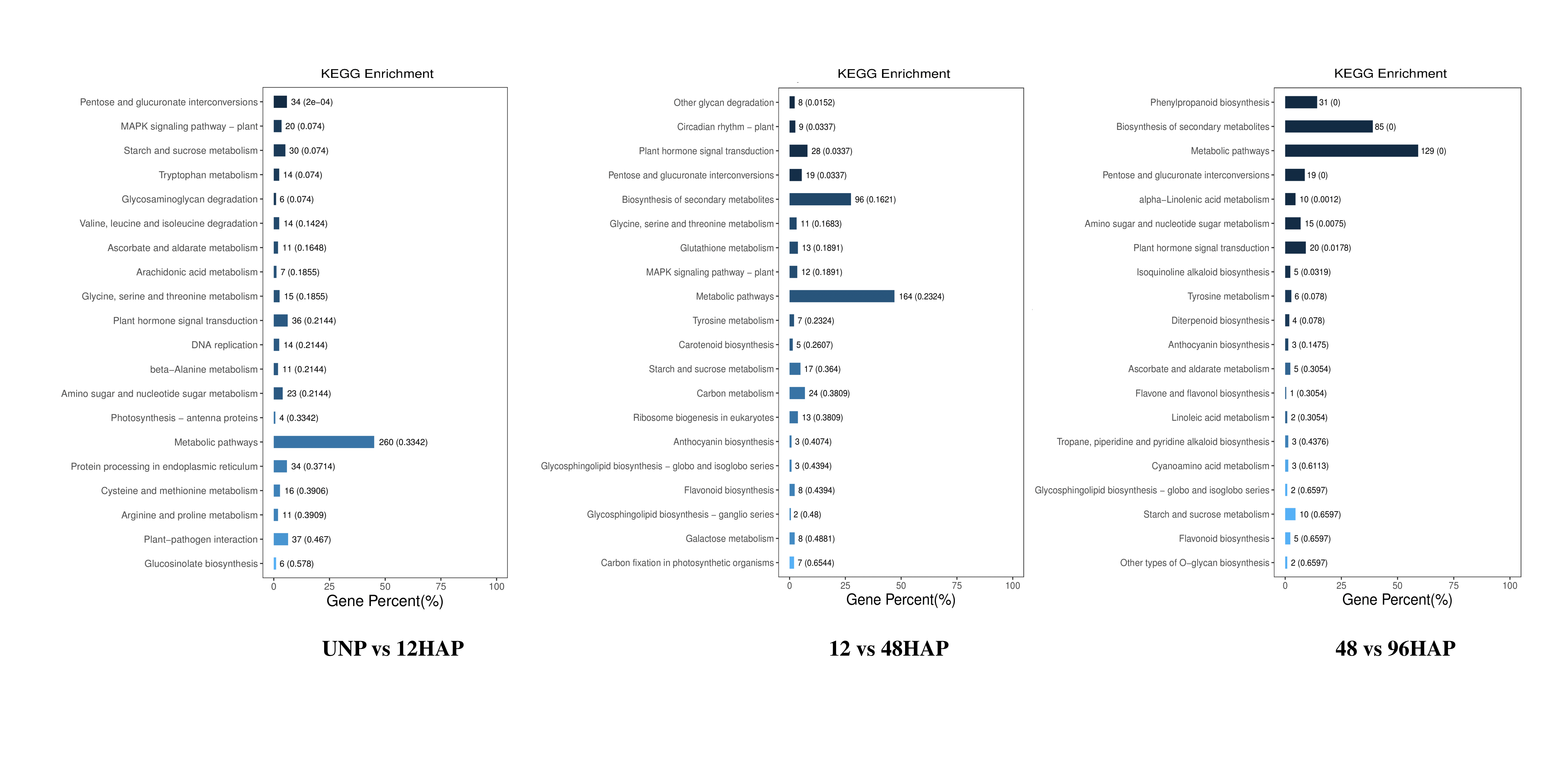
**Figure S2**: KO enrichment analysis DEGs in pairwise analysis, values in parentheses () shows q-value of each KO term


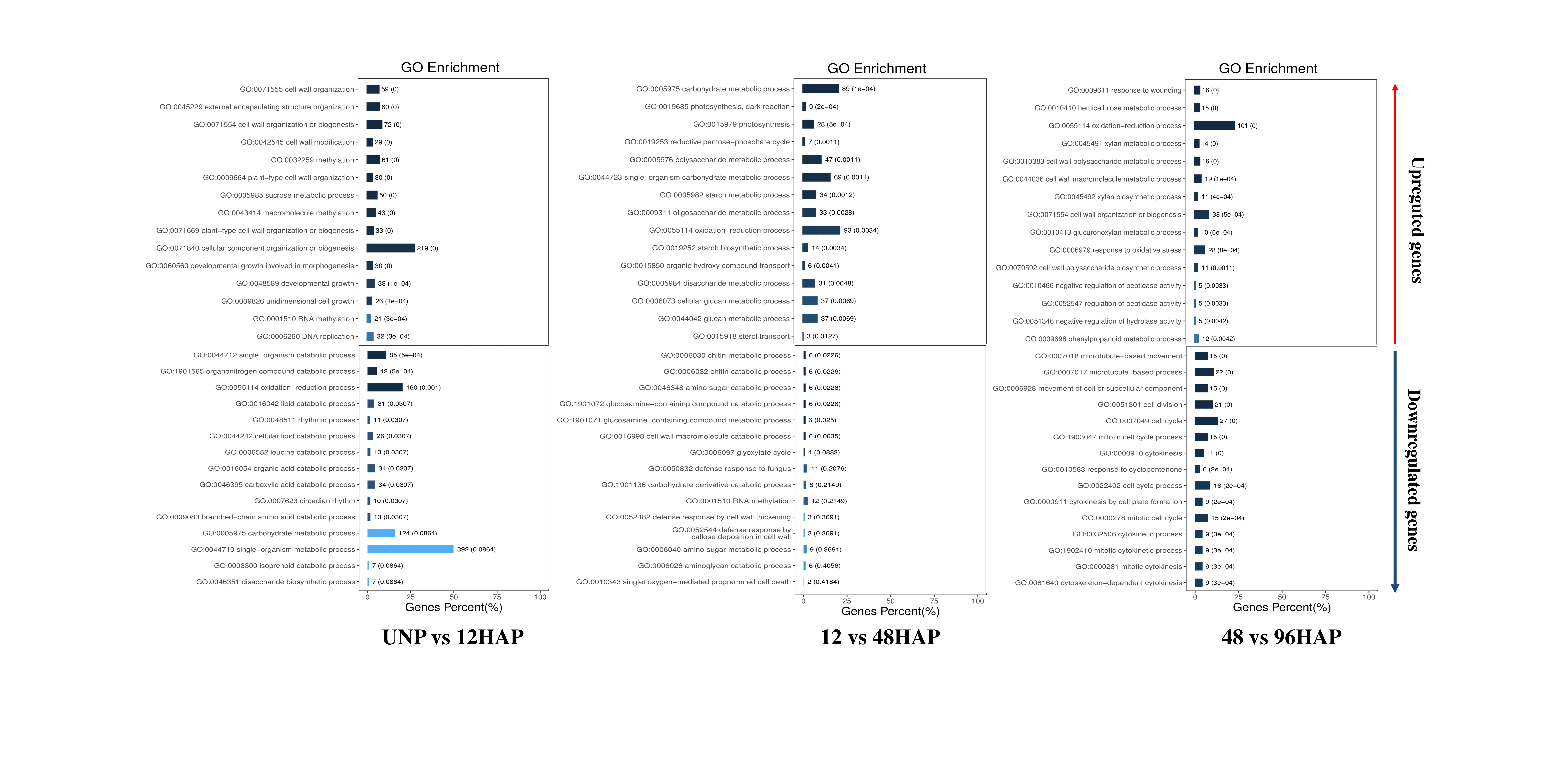


**Figure S3**: GO enrichment analysis of upregulated and down regulated DEGs in pairwise analysis, values in parentheses () shows q-value of each GO term
